# Mono-sensitisation to peanut component Ara h 6: a case series of five children and literature review

**DOI:** 10.1007/s00431-016-2733-7

**Published:** 2016-05-20

**Authors:** J. P. M. van der Valk, M. W. J. Schreurs, R. el Bouch, N. J. T. Arends, N. W. de Jong

**Affiliations:** 1 Department of Internal Medicine, section of Allergology, Erasmus MC, Office Gk 323, P.O. box 2040, 3000CA Rotterdam, The Netherlands; 2 Department of Immunology, Laboratory Medical Immunology, Erasmus MC, Rotterdam, The Netherlands; 3 Department of Paediatric Allergology, Diaconessenhuis Voorburg, Reinier de Graaf Group, Delft, The Netherlands

**Keywords:** Ara h 6, Components, Double-blind placebo-controlled food challenge (DBPCFC), Peanut allergy

## Abstract

Here, we summarise the current clinical knowledge on Ara h 6 sensitisation and clinical relevance of this sensitisation pattern using five illustrative clinical cases. The literature search yielded a total of 166 papers, and an additional relevant article was found by ‘snowballing’. A total of ten articles were considered relevant for this review. Most studies included patients with a sensitisation to Ara h 6 and cosensitisation to Ara h 2. Only three studies showed patients with a mono-sensitisation to Ara h 6. This illustrates that Ara h 6 mono-sensitisation has been neglected in literature. We present a case series of five children with sensitisation to peanut component Ara h 6. Only one of these five patients showed Ara h 8 cosensitivity. Three out of the five children had a positive double-blind placebo-controlled food challenge (DBPCFC), with moderate to strong reactions.

*Conclusion*: A mono-sensitisation to peanut component Ara h 6 is uncommon but can cause severe allergic reactions. Therefore, the determination of sIgE to Ara h 6 is warranted in patients with a suspected peanut allergy, especially in the absence of sensitisation to Ara h 1, 2, 3 and 9.

**Table:** 

**What is known**: • *Peanut allergy is common and can cause severe allergic reactions*. • *The diagnostics of peanut allergy has recently improved with the use of component resolved diagnosis*
**What is new**: • *A mono*-*sensitisation to peanut component Ara h 6 is uncommon*, *but can cause severe allergic reactions* • *Determination of sIgE to Ara h 6 is warranted in patients with a suspected peanut allergy*, *especially in the absence of sensitisation to Ara h 1*, *2*, *3 and 9*

## Introduction

Peanut allergy is common and can cause severe allergic reactions. Even a tiny amount of peanut allergen can induce allergic reactions, including anaphylaxis. The majority of peanut-allergic patients remain allergic to peanuts for the rest of their lives [3]. Diagnosis is traditionally based on the clinical history, sensitisation (skin prick test (SPT) and/or specific IgE (sIgE) and optionally, a double-blind placebo-controlled food challenge (DBPCFC) test [26].

The diagnostics of peanut allergy has recently improved with the use of component-resolved diagnosis (CRD). CRD measures sIgE against individual allergens utilising purified or recombinant allergens. The ImmunoCAP ISAC is an advanced method that detects sIgE for most individual peanut components [9]. More than 13 peanut components have been identified and accepted by the Allergen Nomenclature Subcommittee of the International Union of Immunological Societies (IUIS). The most important and clinically relevant components are Arachis Hypogaea (Ara h) 1, 2, 3, 6, 8 and 9.

Ara h 1, 2 and 3 are seed storage proteins that can induce severe allergic reactions. sIgE to Ara h 2 is the best predictor for a clinically relevant peanut allergy and has high diagnostic accuracy in comparison to other components [14]. Ara h 8 is a pathogenesis-related class 10 (PR-10) protein homologous to the Bet v 1 allergen component of birch pollen and is involved in the cross-sensitisation between pollen and peanut. Allergic reactions due to this cross-reactive protein usually remain limited to oral allergy symptoms. Anaphylaxis due to Ara h 8 has been described only in exceptional cases [11]. Sensitisation to Ara h 9, a lipid transfer protein (LTP), is in most cases accompanied by sensitisation to Ara h 1, 2 and 3[23]. Ara h 9 is a clinically relevant allergen in peanut-allergic patients around the Mediterranean [17, 19].

This systematic review focuses on the clinical relevance and severity of sensitisation to component Ara h 6, a 2S albumin, a conglutin [16]. Little attention is paid in the literature to Ara h 6 in contrast to Ara h 2 sensitisation, which has been investigated far more extensively and, hence, is now generally recognised as predictor for clinically relevant peanut allergy [12, 21, 22]. However, Ara h 6 and Ara h 2 are both 2S albumin storage proteins and approximately 60 % homologous. Ara h 6 and Ara h 2 are comparable in molecular size, amino acid sequence and structure [6, 13, 15, 20].

In this study, we summarise the current clinical knowledge on Ara h 6 sensitisation and describe five relevant cases with four out of five cases showing mono-sensitisation to Ara h 6. We measured clinical relevance of sensitisation to Ara h 6 by performing DBPCFCs.

## Method

### Data sources and literature search

**Fig. 1:**
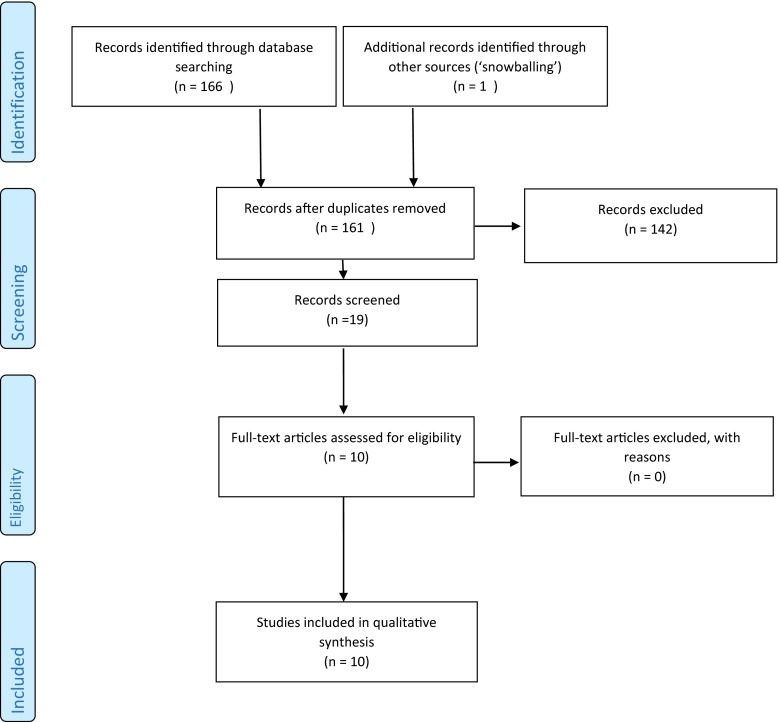
Summary of the search and selection

Our search was performed in line with the methods and procedures of the Preferred Reporting Items for Systematic Reviews and Meta-analyses (PRISMA) guidelines for reporting this systematic review, excluding irrelevant items. The registration number in PROSPERO is CRD42015020451. We used Ovid MEDLINE, EMBASE, Cochrane, Web-of-science, Scopus and Google scholar databases to identify relevant articles by using strings shown in Table 1. We only included studies in English, and there was no restriction on publication date. Mouse models and animal studies were excluded. Initially, all articles on Ara h 6 and Ara h 2 were included because studies on a mono-sensitisation to Ara h 6 were not available. Subsequently, in vitro studies, biochemical studies, studies using skin prick tests with Ara h 2 and/or Ara h 6 extracts only and studies on dermatitis were excluded. Nine of 166 articles identified in our initial database search and one article found by ‘snowballing’ were considered relevant for this systematic review. The literature screening was performed by one author, and the included studies were checked by a second author. This literature selection procedure is shown schematically in Fig. 1.

**Table 1:** Search stings used to identify relevant articles

Embase	(((ara OR nara OR mara) AND ((h2 AND h6) OR (h-2 AND h-6) OR ‘h 2 6’ OR ‘h2 6’)) OR (arah2 AND arah6) OR (arah-2 AND arah-6) OR arah2-6 OR arah-2-6):ab,ti
PubMed	(((ara[tiab] OR nara[tiab] OR mara[tiab]) AND ((h2[tiab] AND h6[tiab]) OR (h-2[tiab] AND h-6[tiab]) OR h-2-6[tiab] OR h2-6[tiab])) OR (arah2[tiab] AND arah6[tiab]) OR (arah-2[tiab] AND arah-6[tiab]) OR arah2-6[tiab] OR arah-2-6[tiab])
Cochrane	(((ara OR nara OR mara) AND ((h2 AND h6) OR (h-2 AND h-6) OR ‘h 2 6’ OR ‘h2 6’)) OR (arah2 AND arah6) OR (arah-2 AND arah-6) OR arah2-6 OR arah-2-6):ab,ti
Web-of-science	TS = ((((ara OR nara OR mara) AND ((h2 AND h6) OR (h-2 AND h-6) OR “h 2 6” OR “h2 6”)) OR (arah2 AND arah6) OR (arah-2 AND arah-6) OR arah2-6 OR arah-2-6) )
Scopus	TITLE-ABS-KEY((((ara OR nara OR mara) AND ((h2 AND h6) OR (h-2 AND h-6) OR “h 2 6” OR “h2 6”)) OR (arah2 AND arah6) OR (arah-2 AND arah-6) OR arah2-6 OR arah-2-6) )
Google scholar	“ara h 6” “ara h 2”

This review comprises a retrospective cases series of five children with sensitisation to component Ara h 6. These patients were selected from the clinical practice of the Department of Allergology, Erasmus MC in Rotterdam and the Department of Paediatric Allergology, Renier de Graaf Group (RdGG) in Delft, the Netherlands, between 2012 and 2015. Sensitisation to peanut was measured by both SPT and sIgE. Peanut components Ara h 1, 2, 3, 6, 8 and 9 were measured, and finally, clinical relevance was evaluated by performing a peanut DBPCFC test.

A SPT with peanut extract was performed by applying a drop of the allergen extract on the skin of the volar aspect of the forearm. The extract was pierced through the skin barrier with a lancet. The SPTs were performed with peanut extracts, generated as previously described by de Jong et al. [8]. A dilution buffer was used as negative control, and histamine (1 %) was used as positive control. A mean diameter ≥3 mm was considered as positive [8]. For the SPT extracts, the peanuts (raw, not roasted, unsalted organic nuts) were homogenised mechanically, ground with a mortar and pestle, defatted by ether extraction, and subsequently air-dried. This pretreated material was stored at −20 °C until needed for further preparation. The pretreated peanut was defrosted regularly, and a 10 % (*w*/*v*) extract in phosphate-buffered saline (PBS pH 7.4, containing 0.03 % human serum albumin and 0.5 % phenol) was made. For use in SPT, the extract was defrosted an hour before the skin test and mixed [8].

The Immulite 2000 XPi system (Siemens) was used to determine sIgE to peanut extract, based on the principle of solid-phase, two-step and chemilumuniscent immunoassay. A value of >0.35 IE/L is considered as positive. The ImmunoCAP ISAC provided sIgE results for 112 components from more than 51 allergens simultaneously, using only 30-mL serum, and was used to determine sIgE to Ara h 1,2,3,6,8 and 9. The micro-array ISAC 112 (ThermoFisher, Uppsala, Sweden) is a solid-phase fluoro- immunoassay that detects specific IgE antibodies to the proteins fixed on ISAC surface. The technique was performed following the manufacturer’s instructions [9]. The results were measured in ISAC Standardized Units (ISU-E). Values <0.3 are considered negative, 0.3–0.9 are considered as low, 1–14.9 as moderate to high and ≥15 as very high.

Each patient underwent a DBPCFC with masked food containing the suspected allergen in an increasing dose at time intervals of 30 min. Placebo and active challenges were randomly administered on separate days with at least a 1-week interval. Blinding was ensured for the physician, the nurse and the patient, and blinding was broken 24 h after the challenge. The validated and standardised food challenge material used in the DBPCFC was prepared according to the recipe developed by Berber-Vlieg et al. in 2008[27]. Flour of roasted peanuts were used and the food matrix predominantly consisted of wheat, sugar, gingerbread spice mix and coconut. The food challenge test consisted of a six-step incremental doses regime. Upon completion of the challenge test, the child had consumed 1.75, 3.5, 14, 70, 130 and 350 mg protein, equivalent to three peanuts in total. The DBPCFC test was discontinued and considered positive when (1) objective symptoms occurred, or when (2) subjective symptoms re-occurred twice after the same dose of challenge material had been administered, three times consecutively, or (3) when severe subjective symptoms persisted for more than an hour. Placebo reactions were assed according to a protocol previously described by Vlieg-Boertsra et al. [28]. If the child presented with the same symptoms on the placebo as on the verum day, the DBPCFC test was considered as undetermined.

### Relevant clinical papers

The ten relevant clinical studies on Ara h 6 mono-sensitisation or Ara h 6 with cosensitisations are summarised here and presented in Table 2.

**Table 2:** Relevant clinical studies on Ara h 6 and/or Ara h 2 sensitisation

Author *Paper*, *year of publication*	Inclusion criteria	Total included peanut allergic patients	Results DBPCFC	sIgE sensitisation (% of all included patients)
DBPCFC performed				
Peeters, Koppelman et al. *Clinical and Exp. Allergy*, *2007*(25)	Clinical history of peanut allergy SPT ratio ≥0.25 or positive peanut sIgE	30 adults	25 performed 22 positive	22 positive to at least Ara h 2 and Ara h 6 (73.3 %) 5 mono-sensitisation to Ara h 2 (16.7 %) 0 mono-sensitisation to Ara h 6 (-)
Flinterman, Van Hoffen et al. *Clinical and Exp. Allergy*, *2007*(10)	Positive DBPCFC peanut SPT ratio ≥0.25 Positive peanut sIgE	20 children	20 performed 20 positive	16 positive to at least Ara h 2 and Ara h 6 (80 %) 4 mono-sensitisation to Ara h2 (20 %) 0 mono-sensitisation to Ara h 6 (-)
Codreanu, Collignon et al. *Int Arch Allergy Immunol*, *2011*(7)	Positive DBPCFC peanut	166 children	166 performed All positive	149 positive to at least Ara h 2 and Ara h 6 (89.7 %) 10 mono-sensitisation to Ara h 2 (6.0 %) 3 mono-sensitisation to Ara h 6 (1.8 %)
Asarnoj, Glaumann et al. *Int Arch Allergy Immunol*, *2012*(4)	Clinical history of peanut allergy Positive peanut sIgE	5 children	5 performed 5 positive	0 positive to at least Ara h 2 and Ara h 6 (-) 0 mono-sensitisation to Ara h 2 (-) 1 sensitisation to Ara h 6 and Ara h 8 (20 %)
Klemans, Knol et al. *Allergy*, *2014*(13)	DBPCFC peanut between 2002 and 2013 Leftover serum	107 adults	107 performed 65 positive	48 positive to at least Ara h 2 and Ara h 6 (44.8 %) 1 mono-sensitisation to Ara h 2 (0.9 %) 4 mono-sensitisation to Ara h 6 (3.7 %)
Kukkonen, Pelkonen et al. *Allergy*, 2015(18)	Peanut-sensitisation or a high suspicion of peanut allergy	102 children	102 performed 69 positive	*No specification*
Ballmer-Weber, Lidholm et al. *Allergy, 2015*(5)	DBPCFC peanut positive or history peanut allergy	68 children and adults	28 performed	*50 positive to at least Ara h 2 and 6* (*74* %) *0 mono*-*sensitisation to Ara h 6*
No DBPCFC performed				
Agabriel, Ghazouani et al. *Pediatr Allergy Immunol*, *2015*(2)	Clinical history of peanut allergy in last 6 months SPT wheel ≥4 mm and/or positive peanut sIgE	117 children	–	73 positive to Ara h 2 (63 %) 74 positive to Ara h 6 (64 %) *No specification*
Ackerbauer, Bublin et al. *Int Arch Allergy Immunol*, *2015*(1)	History of peanut allergy Positive SPT and/or positive peanut sIgE	33 adults 32 children	–	46 positive to at least Ara h 2 and Ara h 6 (70.7 %) *No specification*
Pedrosa, Boyono-Martinez et al. *Ann Allergy Asthma Immunol, 2015*(24)	History of peanut allergy Positive SPT and positive peanut sIgE	22 children	–	13 positive to Ara h 2 and Ara h 6 (59 %) 4 mono-sensitisation to Ara h 6

The first study by Peeters et al. included 30 peanut allergic patients [25]. The aim of this study was to investigate whether sensitisation to individual allergens Ara h 1, Ara h 2, Ara h 3 and Ara h 6 measured with immunoblots was correlated with clinical severity. Twenty-two patients (73.3 %) were sensitised to Ara h 6 and Ara h 2. There was no Ara h 6 mono-sensitisation observed. The authors concluded that Ara h 2 and Ara h 6 appeared to be the most potent allergens and that sensitisation to these components is indicative for severe allergic reactions.

The study by Flinterman et al. measured the sIgE reactivity with immunoblots to major peanut allergens in 20 peanut-allergic patients [10]. A sensitisation to Ara h 2 and Ara h 6 was observed in 16 patients (80 %). None of the patients had a mono-sensitisation to Ara h 6. This study concluded that Ara h 2 and Ara h 6 were the most frequently recognised major peanut allergens in this study.

The study by Codreanu et al. demonstrated a sensitisation to Ara h 2 and Ara h 6 measured with ImmunoCAP in 149 of the in total 166 peanut-allergic patients (89.7 %) [7]. Ara h 6 mono-sensitisation was observed in three patients (1.8 %).

The cases series published by Asarnoj et al. demonstrated one patient with a sensitisation to Ara h 6 and Ara h 8 measured with ImmunoCAP [4]. This patient developed a grade II anaphylaxis during a food challenge test.

A study with 107 peanut-allergic patients was performed by Klemans et al. [13]. The aim of this study was to assess the diagnostic value of sIgE to Ara h 6. This study showed 48 patients (44.8 %) sensitised to Ara h 2 and Ara h 6 and 4 patients (3.7 %) mono-sensitised to Ara h 6 measured with ImmunoCAP ISAC. The authors concluded that the diagnostic value of sIgE to Ara h 6 on the population level was as good as sIgE to Ara h 2.

Kukkonen et al. performed a study in 102 children with a peanut-sensitisation or a high suspicion of peanut allergy [18]. Peanut components to Ara h 1, 2, 3, 6, 8 and 9 sIgE was measured with ImmunoCAP ISAC, and a food challenge test was performed. There was no specification as to how many patients were sensitised for the different components. However, they concluded that Ara h 2 and Ara h 6 sensitisation was associated with severe reactions.

The study by Ballmer-Weber et al. included 68 children and adults with a positive DBPCFC peanut or a history of peanut allergy [5]. Of these 68 peanut allergic patients, 50 patients were sensitised to Ara h 2 and Ara h 6 measured with ImmunoCAP. Mono-sensitisation to Ara h 6 was not demonstrated. Therefore, the authors concluded that Ara h 6 appears not to be an essential component in the diagnostics of peanut allergy.

The study by Agbriel et al. aimed to evaluate peanut CRD as a diagnostic and prognostic method in 117 children [2]. Seventy-four patients (64 %) were sensitised to Ara h 6 measured with ImmunoCAP ISAC (64 %). The conclusion of this study was that sensitisation to Ara h 6 and Ara h 2 were shown to be the best predictors of peanut allergy in their study population.

Ackerbauer et al. performed a study on 65 peanut allergic patients [1]. Sensitisation patterns against peanut allergens Ara h 1, 2, 3, 6, 8 and 9 were measured with ImmunoCAP ISAC. Forty-six patients (70.7 %) were sensitised to Ara h 2 and Ara h 6. The authors concluded that the majority of symptomatic peanut-allergic patients were sensitised to Ara h 2 and/or Ara h 6.

Finally, Pedrosa et al. studied 22 children with a history of peanut allergy and a positive SPT and positive sIgE peanut. The authors demonstrated with ImmunoCAP ISAC that 13 children were sensitised to Ara h 2 and Ara h 6 and 4 were mono-sensitised to Ara h 6. The conclusion of this study was that the measurement of sIgE to Ara h 2 in combination with Ara h 6 improves the diagnosis of peanut allergy [24].

### Case series

Below, we describe the case studies for the five individual children. The results of SPT peanut, sIgE peanut and peanut components Ara h 1, 2, 3, 6, 8 and 9 and the outcome of DBPCFCs with peanut of the five children are summarised in Table 3.

**Table 3:** SPT peanut (mm), sIgE peanut (IE/L), ISAC peanut components (ISU/E) and DBPCFC peanut results

Patient	1	2	3	4	5
SPT peanut	13	15	8	7	9
sIgE peanut	2.1	5.8	4.1	14.0	6.1
Ara h 1	Neg	Neg	Neg	Neg	Neg
Ara h 2	Neg	Neg	Neg	Neg	Neg
Ara h 3	Neg	Neg	Neg	Neg	Neg
Ara h 6	11.2	7.83	2.32	0.58	2.23
Ara h 8	3,3	Neg	Neg	Neg	Neg
Ara h 9	Neg	Neg	Neg	Neg	Neg
DBPCFC	Urticarial skin lesions, abdominal pain	Itching mouth, abdominal pain, headache	Neg	Nausea and abdominal pain, listless behaviour	Neg

#### Patient 1

A 9-year-old atopic boy was referred to the Erasmus MC’s Department of Allergology because of his cow’s milk allergy. He also showed a peanut and tree nut sensitisation. sIgE to peanut was positive (2.1 IE/L), and the SPT with peanut extract was positive (13 mm). He never consumed peanuts. A DBPCFC peanut was subsequently performed. During the challenge, he experienced no symptoms during the placebo day. On the verum day, he had mild oral itch after each incremental dose, which disappeared quickly and did not worsen during the test. After the last dose he had some abdominal pain. About 4 h after the last part, the symptoms worsened and he developed extensive urticarial skin lesions, associated with abdominal pain. These symptoms disappeared several hours after anti-histamine treatment. ImmunoCAP ISAC revealed a sensitisation to peanut component Ara h 6 (11.2 ISU-E). In addition, Ara h 8 sensitivity was observed (3.3 ISU-E), most likely caused by cross-sensitisation due to primary Bet v 1 (PR-10) sensitisation (56 ISU-E). The DBPCFC was judged as positive and the patient was advised to avoid peanuts.

#### Patient 2

A 15-year-old boy was referred to the Department of Paediatric Allergology RdGG for his peanut allergy. He had experienced a severe anaphylactic reaction after the consumption of peanuts at the age of 10. The symptoms included angioedema of the lips and tongue, hoarseness and vomiting. sIgE to peanut was positive (5.8 IE/L), and the SPT with peanut extract showed a positive result (15 mm). ImmunoCAP ISAC demonstrated an Ara h 6 mono-sensitisation (7.83 ISU-E). The DBPCFC showed no symptoms on the placebo day. On the verum day, after step 3, the patient complained of an itching mouth. Step 3 was repeated and gives no problems, but after step 5, the patient experienced severe persistent abdominal pain for more than an hour and tiredness. The DBPCFC was terminated prematurely, and the patient was treated with anti-histamine, which reduced the symptoms slightly. Once home, the boy experienced a headache and felt tired. The DBPCFC was judged as positive, and the patient was advised to avoid peanuts.

#### Patient 3

A 9-year-old boy was referred to RdGG’s Department of Paediatric Allergology for his allergies. This boy had experienced an allergic reaction after consumption of peanuts at the age of 3 and 5. The allergic reaction at both ages included a swollen and sore throat, difficulty with breathing and angioedema of the eyes. SPT with peanut extract and sIgE peanut were both positive (8 mm and 4.1 IE/L, respectively). In ImmunoCAP ISAC, a mono-sensitisation was measured to component Ara h 6 (2.32 ISU-E). A DBPCFC was performed: the patient showed mild symptoms on the placebo day and milder symptoms on the verum day. The DBPCFC was judged as negative, and the boy was allowed to introduce peanut in his diet.

#### Patient 4

A 13-year-old boy was referred to the Department of Paediatric Allergology, RdGG, proved to be sensitised to tree nuts and peanuts with unknown clinical relevance. SPT with peanut was positive (7 mm), and sIgE to peanut was also positive (14.0 IE/L). ImmunoCAP ISAC demonstrated an Ara h 6 mono-sensitisation (0.58 ISU-E). A DBPCFC was performed. The boy showed no symptoms on the placebo day, but after step 3 on the verum day, he experienced severe subjective symptoms as nausea, abdominal pain and listless behaviour for more than an hour. These symptoms disappeared after treatment with anti-histamine and the boy was sent home asymptomatic. The DBPCFC was judged as positive and the patient was advised to avoid peanuts.

#### Patient 5

A 7-year-old boy was referred to Erasmus MC’s Department of Allergology because of his food allergies. He was sensitised to peanut with unknown clinical relevance. SPT with peanut extract and sIgE to peanut were both positive (9 mm and 6.1 IE/L, respectively). ImmunoCAP ISAC measured a mono-sensitisation to component Ara h 6 (2.23 ISU-E). A DBPCFC was performed and judged as negative, and therefore, the child was allowed to introduce peanuts to his diet.

## Discussion

Our literature search demonstrated that there is little information available on Ara h 6 mono-sensitisation. Therefore, the importance of Ara h 6 sensitisation is difficult to define. Three studies on peanut allergy demonstrated patients with mono-sensitisation. However, studies selected for this review demonstrated that Ara h 2 and Ara h 6 were the most frequently recognised major peanut allergens [1, 10].

Our series of relatively unique cases confirms that patients with a mono-sensitisation to peanut component Ara h 6 do indeed exist. Moreover, this mono-sensitisation may cause moderate to severe allergic reactions. Three out of five children showed a clinically relevant Ara h 6 mono-sensitisation, confirmed by a DBPCFC. These reactions consisted of extensive urticarial skin lesions, headaches, persisting abdominal pain and nausea. In a case report by Asarnoj et al., a severe allergic reaction (anaphylaxis) due to predominant Ara h 6 sensitisation was described; however, the patient was also sensitised to Ara h 8[4]. This was also the case for patient 1 in our cases series. This patient was also sensitised to Ara h 8, most probably due to primary Bet v 1 (PR-10) sensitisation. As a result, this patient is officially not mono-sensitised to Ara h 6. However, Ara h 8 is not a peanut storage protein family member, and we consider Ara h 8 to be sensitisation with mild clinical relevance. In the context of primary sensitisation to peanut storage proteins, we consider this patient to be mono-sensitised to Ara h 6.

Although Ara h 6 sensitisation may apparently cause moderate to severe allergic reactions, it is hardly recognised in clinical practice. The lack of readily available test systems for sIgE against Ara h 6, in addition to the relatively expensive and unique ImmunoCAP ISAC system, probably contributes to this. However, an overlooked Ara h 6 mono-sensitisation may cause harmful situations during a food challenge or peanut introduction at home. Therefore, the importance of the Ara h 6 sIgE determination should not be underestimated. In recent years, most researchers have focused on the Ara h 2 peanut component and found that sensitisation is highly predictive of a severe clinical peanut allergy. In addition to Ara h 2, Ara h 6 sensitisation coexists in many peanut-allergic patients [13]; however, mono-sensitisation to Ara h 6 also exists, as this study clearly demonstrates. This warrants increased attention being paid to patients with Ara h 6 mono-sensitisation.

## Conclusion

A mono-sensitisation to peanut component Ara h 6 is uncommon, but in some cases clinically relevant as it can cause severe allergic reactions, including anaphylaxis. Therefore, the determination of sIgE to Ara h 6 is warranted in patients with a suspected peanut allergy, especially in the absence of sensitisation to Ara h 1, 2, 3 and 9.

## Abbreviations

Ara h: Arachis hypogaea
CRD: Component-resolved diagnosis
DBPCFC: Double-blind placebo-controlled food challenge
IUIS: International Union of Immunological Societies
LTP: Lipid transfer protein
PBS: Phosphate-buffered saline
PR-10: Pathogenesis-related class 10
sIgE: Specific IgE
SPT: Skin prick test
